# Successful Treatment of a Patient With Multiple-Line Relapsed Extensive-Stage Small-Cell Lung Cancer Receiving Penpulimab Combined With Anlotinib: A Case Report

**DOI:** 10.3389/fonc.2022.846597

**Published:** 2022-03-07

**Authors:** Zibo Zhang, Yujun Li, Yan Dong, Jia Li, Bin Zhang, Chunxia Zhang, Xiaonan Cui

**Affiliations:** Department of Oncology, The First Affiliated Hospital of Dalian Medical University, Dalian, China

**Keywords:** penpulimab, anlotinib, ICI rechallenge, small-cell lung cancer, case report

## Abstract

Small-cell lung cancer (SCLC) is a highly malignant, rapidly developing group of diseases with poor biological behavior. Most patients have extensive-stage SCLC (ES-SCLC) when they are first diagnosed. Standard chemotherapy is prone to relapse in a short period of time, and the patients’ median overall survival (OS) can reach only 13 months when chemotherapy is given in combination with PD-L1 inhibitors. To date, no studies have verified the efficacy and safety of the composite treatment of ES-SCLC with penpulimab and anlotinib despite some recognized data and advantages related to this regimen. Penpulimab, a novel PD-1 inhibitor with an IgG1 subtype, has a structural modification of the Fc segment which can prevent the immune cells from being phagocytosed or killed and can steadily avoid tumor immune escape. This case report describes a 71-year-old man who had ES-SCLC for 7 years which progressed after receiving standard systemic chemotherapy combined with radiotherapy. The third-line treatment of four cycles of anlotinib and carilizumab was discontinued because of grade 2 immune-related pneumonia despite the efficacy being evaluated as stable disease. After maintaining 22 months of progression-free survival, the patient relapsed and switched to a safer regimen of penpulimab combined with anlotinib to continue the treatment for four cycles. Partial response evaluation was confirmed twice, and the patient remained in good general condition. The combination of penpulimab and anlotinib can positively regulate the therapeutic effect by simultaneously acting on the tumor microenvironment and promoting blood vessel normalization. In general, this case provides support for the successful possibility of a rechallenge with immune checkpoint inhibitors, the better clinical efficacy of cross-line therapy with anlotinib, and the drug safety of penpulimab, suggesting a beneficial therapy for the clinical treatment of ES-SCLC.

## Introduction

Although extensive-stage small-cell lung cancer (ES-SCLC) is very sensitive to initial treatment, with a tumor remission rate of 60–80%, most patients still experience relapse or drug resistance after initial treatment. SCLC patients have a median overall survival (OS) of only 4–5 months after further chemotherapy (1, 2), and their general prognosis is poor (3). Although the efficiency of treatment depends largely on the time interval between the end of the initial treatment and relapse, an individualized selection of effective later-line treatment options significantly relieves symptoms.

SCLC can produce a better immune response with immune checkpoint inhibitors (ICIs) because of its high mutational burden and immunogenicity. Therefore, the combination of immunotherapy and chemotherapy can significantly increase anti-tumor efficacy and improve prognosis compared to chemotherapy alone (4). Penpulimab (trade name: Anico) is a new type of recombinant humanized anti-PD-1 monoclonal antibody (mAb) with a special subtype of IgG1 structure that is relatively stable. A modified Fc segment and an optimized Fab segment can silence the Fc effect by preventing the phagocytosis or killing of immune cells and reducing fever and infusion reactions. Penpulimab, a novel mAb against PD-1 with anIgG1 subtype, not only enhanced the efficacy of immunotherapy but also greatly improved the safety of the drug. In addition, anlotinib is an oral multi-target tyrosine kinase inhibitor that selectively inhibits vascular endothelial growth factor receptor, fibroblast growth factor receptor, platelet-derived growth factor receptor, c-Kit, and c-Met (5, 6). The ALTER1202 study (7, 8) showed that anlotinib could be a third-line standard treatment for patients with ES-SCLC for whom chemotherapy failed.

In recent years, many positive results have been achieved with anti-PD-1 ICIs combined with anti-angiogenic-targeting regimens, such as lenvatinib plus pembrolizumab for hepatocellular carcinoma (9) and atezolizumab plus bevacizumab for renal cell carcinoma (10). In 31 patients evaluated based on the RECIST 1.1 criteria, the first-line treatment of hepatocellular carcinoma with anlotinib in combination with penpulimab achieved an overall response rate (ORR) of 31%, a disease control rate (DCR) of nearly 83%, and a median progression-free survival (PFS) of 8.8 months, which was comparable to the efficacy of similar anti-angiogenic therapy and immunotherapy combinations. Adverse effects were manageable, and the safety profile was deemed satisfactory (11). Similarly, studies on the treatment of ES-SCLC have repeatedly reported the clinical benefits of this drug combination approach. In a single-arm, open, phase Ib dose exploration study of the treatment of advanced solid tumors with TQB2450 (PD-L1 inhibitor) and allotinib among six patients with SCLC, four cases showed that the treatment had a partial response (PR) efficacy (12). In a phase 2 study of the second-line treatment of SCLC (13), it was observed that the ORR of patients with SCLC who received carilizumab combined with apatinib as a second-line treatment reached 33.9%, and the median OS was 8.4 months. It is worth noting that the median OS was even 8.0 months in resistant patients, suggesting that the combination of ICIs and antiangiogenic drugs is a promising therapeutic strategy for recurrent and advanced SCLC with increased clinical recognition.

As an mAb against PD-1 with a new IgG1 subtype, penpulimab has only been approved for marketing in China. Currently, there are only recurrent or refractory indications for Hodgkin’s lymphoma. There are no published or relevant treatment data or case reports for penpulimab as a treatment for SCLC; therefore, its therapeutic efficacy is unclear. The first experimental application of penpulimab for patients suffering from recurrent SCLC and immune-related pneumonia due to other mAbs is shown in this case, along with successful immune rechallenge treatment. The patient achieved continuous remission, and the therapeutic effect was evaluated as PR compared with the initial treatment. Moreover, the patient’s general condition remained good without serious adverse reactions.

## Background

**Figure 1:**
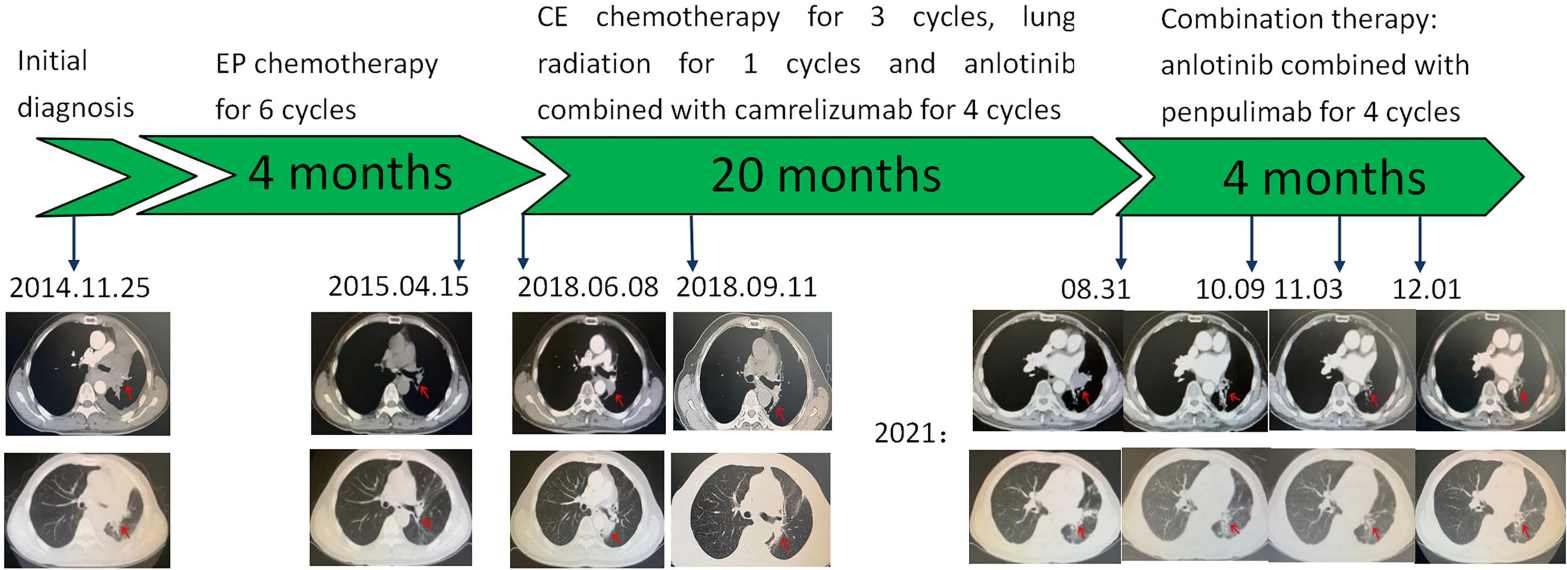
Graphic timeline of the case.

A 71-year-old man was admitted to the hospital for “repeated cough and sputum” in early November 2014. He had a BS of 1.96 m^2^, Eastern Cooperative Oncology Group score of 1, atrial fibrillation for more than 10 years, and no smoking history or family history. The chest CT (November 25, 2014) (**Figure 1**) revealed the following: left pulmonary central cancer, obstructive atelectasis in the left upper lobe, obstructive pneumonia, nodules in the left lower lobe, and left hilar lymph node metastasis. The left lower pulmonary veins might have been involved, including multiple millet lesions in the right lung and pleural fluid on the left side. No obvious signs of bone metastasis were observed on bone ECT or PET-CT. The histopathological examination of the fibreoptic bronchoscopy and immunohistochemistry samples revealed heterogeneous cell clusters in the diseased tissue: CD56 (NK-1) (+), CgA (minority +), and TTF-1 (+). The results of the chest CT, histopathology, immunohistochemistry, bronchial brush tablets, and lavage fluid base provided sufficient evidence for the diagnosis of left SCLC, staged as ES.

The patient received chemotherapy with the etoposide and cisplatin for six cycles from December 6, 2014 to March 26, 2015, and lung radiotherapy and prophylactic brain irradiation were continued since April 25, 2015. In early June 2018, the patient suddenly developed a cough and expelled blood-stained sputum. According to the subsequent chest CT (June 8, 2018) (**Figure 1**), there were multiple blurred patches and striped shadows in the lung tissue near the mediastinum of the left lung and the left hilar descending aorta. A lump of about 3.2 × 2.3 cm was seen on the side, which was considered malignant. There were multiple miliary shadows in the left lung and irregular nodules in the lower left lobe, with a diameter of approximately 0.6 cm each. Considering the recurrence of the patient’s condition, he was administered the regimen with carboplatin and etoposide for three cycles. After that, the lung lesion was reduced by 25% so that the curative effect was evaluated as SD, and local radiotherapy was continued for one cycle. On November 1, 2019, the patient coughed up blood again. After four cycles of treatment with anlotinib and carilizumab, the symptoms improved, and the efficacy was evaluated as SD. However, the patient developed grade 2 immune-related pneumonia, leading to the discontinuation of this regimen. On August 31, 2021, the patient was reexamined *via* lung CT after “coughing up sputum and hemoptysis for 1 week” (**Figure 1**). Multiple nodules and masses were identified in the lower lobe of the left lung; new larger nodules were apparent in the hilar area, approximately 3.8 × 2.3 cm in size. Multiple miliary foci were observed in both lungs. Left pleural effusion and thickened left pleura were also observed. There were multiple small lymph nodes in the left supraclavicular fossa with a shorter diameter of 0.3 cm. The patient’s condition recurred. For further treatment, an unprecedented and experimental combination therapy with penpulimab and anlotinib was applied for two cycles. The chest CT (October 09, 2021) (**Figure 1**) revealed the following: The size of the mass in the left lower hilar area was about 3.5 × 2.5 cm and was reduced when compared with the chest CT on August 31, 2021. Small nodules and strips were also seen in the left lower lobe; the largest was about 0.7 × 0.3 cm, and the rest were similar to those previously described. The effect of PR was evaluated based on the examination of the images. After receiving the combination treatment for two cycles, a re-examination *via* chest CT (November 03 and December 06, 2021) (**Figure 1**) showed that the patient’s condition was stable, and the curative effect assessment of lesions showed a shrinking trend within the SD range. In summary, the effect was significant, and so far, no immune-related adverse reactions occurred after four cycles of treatment, which further proved the considerable clinical efficacy and drug safety of this regimen. Timeline of the treatment was shown in **Table 1**.

**Table 1:** Timeline of the treatment.

Time	Major medical examination	Diagnosis or disease evaluation	Treatment
2014.11.25	Chest CT, whole-body bone scan, positron emission tomography–computed tomography, fiber bronchoscopy	Small-cell lung cancer of the left lung at ES stage	Puncture of the lung lesions
2014.12.06–2015.03.26	–		(Etoposide: 200 mg on day 1, day 2, and day 3 + 100 mg on day 4 + cisplatin: 60 mg on day 1 and day 2) for 6 cycles
2015.04.15	Chest CT	Partial response	
2015.04.25	–		Lung radiotherapy and prophylactic cranial radiotherapy
2018.06.08	Chest CT	Local relapse	
2018.06.09–08.12	–		(Etoposide: 200 mg on day 1, day 2, and day 3 + 100 mg on day 4 + carboplatin: 500 mg on day 1) for 3 cycles
2018.09.11	Chest CT	Stable disease	Lung radiotherapy
2019.11.01	Chest CT	Local relapse	
2019.11.02–2020.02.05	–		(Anlotinib: 8 mg from day 1 to day 14 + camrelizumab: 200 mg on day 1) for 4 cycles
2021.08.31	Chest CT	Local relapse	Anlotinib: 8 mg from day 1 to day 14 + penpulimab: 200 mg on day 1
2021.10.09	Chest CT	Partial response	Anlotinib: 8 mg from day 1 to day 14 + penpulimab: 200 mg on day 1
2021.11.03	Chest CT	Stable disease	Anlotinib: 8 mg from day 1 to day 14 + penpulimab: 200 mg on day 1
2020.12.06	Chest CT	Stable disease	Anlotinib: 8 mg from day 1 to day 14 + penpulimab: 200 mg on day 1

## Discussion

SCLC is a highly aggressive neuroendocrine tumor with high malignancy, easy metastasis, and rapid progression. Based on the Impower133 and Caspian trials (14, 15), the US Food and Drug Administration recommended atezolizumab or durvalumab combined with platinum as the first-line treatment option for ES-SCLC. Despite the high response rate to initial platinum therapy, almost all patients with ES-SCLC relapse after a short-term treatment with a poor prognosis. Topotecan is a currently approved second-line standard treatment, and navuluzumab or palolizumab can also be used for the treatment of recurrent SCLC. However, the National Comprehensive Cancer Network recommended subsequent systemic and palliative symptomatic treatment after the failure of first- or second-line treatment, suggesting that there is no standard treatment recommendation. Most patients will progress after receiving two or more previous treatment regimens, and there are several limitations with third- and later-line treatment options for patients who cannot receive effective drug treatment, thus affecting their OS. The PFS of this patient after frontline platinum-containing chemotherapy was longer, which suggested that the patient had better drug sensitivity.

Anlotinib inhibits tumor growth through anti-tumor angiogenesis and controls tumor cell proliferation and metastasis (16). In the ALTER-1202 study, anlotinib brought better survival benefits to patients receiving third- and subsequent-line treatment options for SCLC; their median PFS was extended by 3.4 months (hazard ratio, HR: 0.19), and their median OS was prolonged from 4.9 to 7.3 months (HR: 0.53) when compared with the placebo. According to the ALTER-1202 study, the Guidelines for the Diagnosis and Treatment of SCLC in Chinese Society Clinical Oncology recommended anlotinib as a standard choice for the third-line treatment of SCLC. In 2019, the National Medical Products Administration also approved the use of anlotinib for SCLC, providing a standard third-line therapy for patients with SCLC in China.

Other mAbs against PD-1 currently on the market all use IgG4 subtypes, while IgG1 is only applied in penpulimab. MAbs with IgG4 subtypes can give rise to poor stability, Fc–Fc interactions, and antibody drug aggregation and can combine with anti-tumor-specific IgG1 to inhibit natural IgG1 performance and promote tumor immune escape. By comparison, antibodies with IgG1 subtypes are more stable, which can reduce the likelihood of drug aggregation and prevent tumor immune escape. In addition, most of the listed mAbs against PD-1 are unmodified, leading to a reduction in immune cells and affecting the anti-tumor immune response and IL-8 release. In the case of penpulimab, genetic engineering is used to carry out structural modifications to prevent immune cell destruction and phagocytosis, decreasing the release of IL-8 and enhancing the curative effect. Based on the currently available clinical data, there had not been any comparative studies of two mAbs against PD-1. In the AK105-201 study, penpulimab was applied to the treatment of relapsed and refractory classical Hodgkin’s lymphoma; the ORR was defined as the primary endpoint and reached 89.4%, and all patients had an OS of 18 months. Remarkably, the incidence of grade 3 adverse events in patients receiving parimizumab was only 4.3%, and there was no grade 4 to 5 immune-related adverse event (irAE) compared with the first-generation PD-1 (17).

In this case, the patient was diagnosed with ES-SCLC upon first presentation, with a disease course of up to 7 years. After four cycles of third-line treatment with anlotinib and carilizumab, the disease was evaluated as SD, but treatment was discontinued because of grade 2 immune-related pneumonia. At the end of August 2021, the patient’s lung lesions recurred. Since PFS was maintained for 22 months after anlotinib and carilizumab administration, the patient was initially judged to be someone who could continue to benefit from immunotherapy. Given that the patient’s immune-related pneumonia returned to level 1, immunotherapy was reconsidered. An observational, cross-sectional, pharmacovigilance cohort study showed that about 28.8% of initial irAEs reoccurred upon rechallenge treatment with ICIs (18). For patients who consider resuming ICI treatment, it is necessary to reduce the possibility of irAE occurrence, leading to discontinuation. Therefore, the original therapy was replaced with a safer ICI, which, in this case, was penpulimab. After four cycles of treatment with the combined regimen of penpulimab and anlotinib, efficacy was assessed as PR when compared with the baseline. The patient benefited from the therapy continuously, without further adverse effects such as immune-related pneumonia. Therefore, this case not only proves the superior safety of penpulimab but also shows that rechallenge in immunotherapy with ICIs and the trans-line treatment with anlotinib are still effective, bringing great clinical benefit to patients with ES-SCLC. Compared to the successful approval of PD-L1 ICIs, two PD-1 drugs, nivolumab and pembrolizumab, were withdrawn by the FDA for the treatment of SCLC in 2020 and 2021, respectively, due to their limited benefits. In this case, a PD-1 inhibitor combined with anlotinib might improve the tumor remission rate of PD-1 ICIs and is a novel idea to try for SCLC treatment.

In recent years, data on the safety and effectiveness of restarting ICIs after immunotherapy has been interrupted by the presence of many irAEs. A retrospective study indicated that 68 patients with non-small-cell lung cancer (NSCLC) who were administered an ICI stopped their treatment due to irAEs, with only 38 patients then resuming treatment. Subsequently, 18 patients (48%) did not experience irAE recurrence, and the irAEs experienced thereafter were only mild to moderate (12/20, 60%). ICI rechallenge in patients who discontinued treatment due to irAEs may have potential benefits. In the KEYNOTE-010 trial, patients with NSCLC who received pembrolizumab retreatment had an ORR of up to 42.9%; another large European retrospective analysis showed that patients who received an ICI rechallenge had a median OS of between 15.0 to 18.4 months (19, 20). Based on clinical experience, this patient may have sustained an immune benefit and received a successful immune rechallenge therapy. This was achieved by switching to the safer penpulimab after the patient developed grade 2 immune-associated pneumonia while on carilizumab, suggesting that the choice of drug for immune rechallenge could be a break from conventional therapy with the original drug (21).

The main reason for the limited benefit of immunotherapy in patients with SCLC is the lack of biomarkers to predict its efficacy and toxicity. Based on a previous classification, Gay et al. (22) analyzed the RNA sequence data of 81 SCLCs to classify SCLC into four transcriptionally distinct subgroups: ASCL1/SCLC-A, NEUROD1/SCLC-N, POU2F3/SCLC-P, and SCLC-inflamed (SCLC-I). The vast differences in the immune microenvironment of different subtypes of SCLC (23), based on the phenotypic molecular expression of the SCLC-I subtype, showed that it may have a higher response to immunotherapy. Additionally, trends were observed in the follow-up analysis of the IMpower133 study, providing an advantage for SCLC in immunotherapy population selection as potential biomarkers and related mechanisms provide strong evidence for this study. It seems possible to declare that the treatment of SCLC has entered the era of precision therapy (24). Therefore, the main limitation of this study is that early judgment of the immune benefit for patients comes from clinical experience after the assessment of efficacy. We hope that future universal molecular typing of SCLC can pre-screen more populations, though this requires more researchers to conduct more in-depth and extensive research.

## Concluding Remarks

In summary, our case further demonstrates the efficacy and safety of penpulimab combined with anlotinib for the later-line treatment of ES-SCLC, and two regimens of different immunological drugs combined with an anti-vascular-targeting agent achieved ideal survival benefits. However, different outcomes in safety suggest that the selection of immune agents in combination therapy may be a key factor affecting the treatment outcome. After the patient developed grade 2 immune-related pneumonia, choosing the safer penpulimab as a rebooted ICI and combining it with the cross-line therapy of anlotinib improved the survival and did not lead to the development of any irAEs. In summary, this combination is a good treatment method for patients with ES-SCLC. It is expected that, with the continuous development of oncology medicine, relevant clinical trials can be conducted to obtain more scientific and rigorous data to verify these findings in the near future.

## Data Availability Statement

The original contributions presented in the study are included in the article/supplementary material. Further inquiries can be directed to the corresponding authors.

## Ethics Statement

Written informed consent was obtained from the individual(s) for the publication of any potentially identifiable images or data included in this article.

## Author Contributions

CZ and XC contributed to the conception and design and provided administrative support. ZZ provided necessary information. YL and ZZ took charge of the collection and assembly of data, conducted the disease analysis, provided the summary. All authors contributed to the article and approved the submitted version.

## Funding

This work was supported by the Wu Jieping Medical Foundation (320.6750.2021-16-6).

## Conflict of Interest

The authors declare that the research was conducted in the absence of any commercial or financial relationships that could be construed as a potential conflict of interest.

## Publisher’s Note

All claims expressed in this article are solely those of the authors and do not necessarily represent those of their affiliated organizations, or those of the publisher, the editors and the reviewers. Any product that may be evaluated in this article, or claim that may be made by its manufacturer, is not guaranteed or endorsed by the publisher.
