# Corrigendum: Successful treatment of a patient with multiple-line relapsed extensive-stage small-cell lung cancer receiving penpulimab combined with anlotinib: A case report

**DOI:** 10.3389/fonc.2022.980842

**Published:** 2022-08-02

**Authors:** Zibo Zhang, Yujun Li, Yan Dong, Jia Li, Bin Zhang, Chunxia Zhang, Xiaonan Cui

**Affiliations:** Department of Oncology, The First Affiliated Hospital of Dalian Medical University, Dalian, China

**Keywords:** penpulimab, anlotinib, ICI rechallenge, small-cell lung cancer, case report

In the published article, there was an error in the affiliations. Instead of including “Graduate School of Dalian Medical University, Dalian Medical University, Dalian, China”, all authors should be affiliated with “Department of Oncology, The First Affiliated Hospital of Dalian Medical University, Dalian, China” as above.

In the published article the order of the corresponding authors were incorrect. Instead of “Chunxia Zhang, qm1210@hotmail.com; Xiaonan Cui, cxn23@sina.com”, it should be “Xiaonan Cui cxn23@sina.com Chunxia Zhang qm1210@hotmail.com.

The authors apologize for these errors and state that this does not change the scientific conclusions of the article in any way. The original article has been updated.

## Publisher’s note

All claims expressed in this article are solely those of the authors and do not necessarily represent those of their affiliated organizations, or those of the publisher, the editors and the reviewers. Any product that may be evaluated in this article, or claim that may be made by its manufacturer, is not guaranteed or endorsed by the publisher.

